# Social Media and the Evolution of Vaccine Preferences During the COVID-19 Pandemic: Discrete Choice Experiment

**DOI:** 10.2196/66081

**Published:** 2025-05-28

**Authors:** Robbie Maris, Zack Dorner, Stephane Hess, Steven Tucker

**Affiliations:** 1 Centre for Education Policy and Equalising Opportunities, University College London, 20 Bedford Way, London, WC1H 0AL, United Kingdom, 44 2076792000; 2 Department of Environmental Management, Lincoln University, Lincoln, New Zealand; 3 Choice Modelling Centre, Institute for Transport Studies, University of Leeds, Leeds, United Kingdom; 4 Waikato Management School, University of Waikato, Hamilton, New Zealand

**Keywords:** health information sources, social media, COVID-19, SARS-COV-2, respiratory, infectious, pulmonary, pandemic, vaccination, stated preferences, attitude, perspective, discrete choice experiment, misinformation, global pandemic, sentiment analysis, social listening, public health

## Abstract

**Background:**

Vaccine information and misinformation are spread through social media in ways that may vary by platform. Understanding the role social media plays in shaping vaccine preferences is crucial for policymakers and researchers.

**Objective:**

This study aims to test whether social media use is associated with changes in vaccine preferences during the COVID-19 pandemic in New Zealand, and whether trust in sources of information has a moderating role.

**Methods:**

Our data consist of a balanced panel of 257 web-based respondents in New Zealand in August 2020, October-November 2020, and March-April 2021. We use a novel approach with stated choice panel data to study transitions between different vaccine preference groups. We analyze the associations between these transitions and social media use. We classify respondents as resistant (never chose a vaccine), hesitant (chose a vaccine between 1 and 5 times), and provaccine (chose a vaccine 6 out of 6 times) in each wave of data.

**Results:**

We found a positive or neutral association between social media use and vaccine uptake. Facebook, Twitter (pre-2022), and TikTok users who are provaccine are less likely to become hesitant or resistant. Facebook and Instagram users who are hesitant are more likely to become pro. Some social media platforms may have a more positive association with vaccine uptake preferences for those who do not trust the government.

**Conclusions:**

The paper contributes to the wider literature, which shows social media can be associated with reinforcing both pro and antivaccination sentiment, and these results depend on where individuals get their information from and their trust in such sources.

## Introduction

The COVID-19 pandemic has had substantial global impacts, with over 6 million deaths reported to the World Health Organization to date, alongside other impacts such as 5%‐20% of cases leading to long COVID and widespread economic disruption [1-3]. Vaccination is a vital tool for combating the pandemic, both now and into the future [4]. Despite the clear benefits of vaccination against COVID-19 and other diseases, many people are hesitant or resistant [5-7]. A rapidly emerging body of literature focuses on the characteristics associated with COVID-19 vaccine uptake [7-10]. Indeed, it is important to use data from this period to study what influences vaccination preferences, given the unprecedented global attention on COVID-19 vaccination, the speed of vaccine development, and the potential for future pandemics [11]. Given worries that vaccination hesitancy could be on the rise, it is doubly important to learn from the COVID-19 pandemic [12].

The role of information, who provides that information, and trust in that information have all been shown to be key factors in vaccination uptake [6,13]. Social media platforms are increasingly used as a source of information [14]. The nature of social media means that information sources and types vary greatly, with everything from conspiracy theories to scientific information. Both types of information have been shown to disseminate in a relatively similar manner, with some differences by platform [15,16]. There is evidence that users tend to seek out information that accords with their pre-existing beliefs, regardless of whether it is factual or not [17]. Thus, users end up in echo-chambers reinforcing their own beliefs regarding vaccination and other socially important topics, particularly when issues are controversial [16,18,19]. Indeed, prior work suggests antivaccine sentiment generates more engagement than provaccine sentiment [20] and that exposure to misinformation can decrease intent to vaccinate [12,21,22]. However, there are still relatively few papers that use panel data to investigate the relationship between social media use and vaccine preferences, and our understanding of this relationship is still limited.

A range of studies use survey methods to investigate the association between social media use and willingness to vaccinate against COVID-19. These studies show a mixed picture. Several multi-national studies of European nations and the United States of America show either a negative or nonsignificant association between social media and willingness to vaccinate, depending on the country [23,24]. Similarly, Park et al [8] conducted a survey in the United States and found the lowest vaccine acceptance levels among those who rely on social media for information. In another USA-based survey, Al-Ugdah et al [25] find a positive association between social media users and willingness to vaccinate, but the opposite when no medical or government source of information is used alongside it. Bendau et al [26] found that Germans who report the use of social media to garner information on vaccination have higher acceptance than those who do not. Wang et al [27] report a similar finding in China. These studies all use cross-sectional surveys, but do not have a panel sample recording changes over time.

There are fewer studies using panel surveys, tracking changes in intention to vaccinate and their relationship with social media use. Romer and Jamieson [28] conducted a panel survey in the United States in March and July 2020. They find that social media use is associated with conspiracy beliefs and vaccine resistance, with underlying political ideology playing a key role. Beliefs and preferences around health behaviors were relatively stable over the survey period. In their panel survey, Theocharis et al [29] find that COVID-19 conspiracy beliefs change over time. Twitter reduces the propensity to believe in this conspiracy, whereas other platforms (Facebook, YouTube, Messenger, and WhatsApp) increase these beliefs. They do not look specifically at intention to vaccinate.

In this paper, we investigate whether social media use is positive or negative for COVID-19 vaccination uptake, both on average and in terms of polarization over time. We conducted a web-based stated preferences panel survey of New Zealanders over 3 waves between August 2020 and April 2021, while vaccines were being developed, approved by regulators, and initially rolled out. New Zealand provides an interesting case study due to the high levels of government trust at the time [30-32], Thus, New Zealand has the potential to show a best-case scenario for the impact of social media on vaccine uptake.

We collected data on respondents’ social media use over the previous 6 months, by platform, in the third survey wave. This 6-month period roughly covers the time between the first survey wave and the third. To test for polarization, we model transitions between types of vaccine preferences between waves using a Logit model, to test whether social media use is associated with a change or a reinforcement of earlier choices. We then model the association of social media use with likely vaccine uptake overall (positive or negative on average) using a pooled partial proportional odds (PPO) model with time fixed effects [33]. Finally, we test how trust in government, friends, and family could moderate the effect of platform use. We contribute to the literature by furthering our understanding of the potential role of social media and trust in information on shaping vaccine preferences. Additionally, we demonstrate a novel approach to analyzing stated preference data, we use panel data to explore changes over time, and we examine preferences during the COVID-19 pandemic in New Zealand—an informative context to study the interactions between social media use, trust in information, and vaccine preferences.

## Methods

### Data

We use the New Zealand data from Hess et al [7], which is a web-based stated choice survey on COVID-19 vaccines, undertaken on 20‐29 August, 2020 in the case of New Zealand. Additionally, we repeated the survey for the panel from 26 October to 18 November, 2020 (wave 2) and 23 March to 24 April, 2021 (wave 3). This element of the research design was important to be able to track changes over time. The panel was recruited using the Qualtrics survey company, and was initially representative of the New Zealand population of 18 years and older for age and gender (reweighting as needed for analysis, described at the end of this section). Qualtrics recruited into the survey from a pool of potential New Zealand-based participants who are available for the purpose of collecting representative data in web-based surveys, with a small financial incentive. We checked the survey responses across the panel, and there were no discernible signs of poor quality responses (eg, very short completion time).

The time period of the 3 waves captures a key moment for information gathering and preference formation regarding vaccines. Development and testing were being undertaken during waves 1 and 2. The Pfizer-BioNTech vaccine was approved by the United States Food and Drug Administration in December 2020, with Medsafe in New Zealand following on 3 February 2021. As the only vaccine initially offered in New Zealand, the rollout of Pfizer-BioNTech to a select group had begun by wave 3, with more advanced rollouts underway in other countries.

Additionally, over this time period, trust in the government increased and was high due to their strong lockdown policies, which were successful in eliminating community COVID-19 cases within New Zealand [30-32]. Demonstrating this high support, Prime Minister Jacinda Ardern’s Labor Party won re-election in October 2020, increasing their vote share to the highest ever for a single political party since New Zealand introduced its proportional representation electoral system in 1996, at 50% of the vote.

The main part of the survey is a stated choice survey, using a technique often called a discrete choice experiment [34]. Respondents were presented with 6 hypothetical, but realistic, choices regarding a COVID-19 vaccine. In each choice, respondents were asked to choose their preferred option out of 2 vaccines (each available as paid or free with a wait time) and no vaccine, giving them 4 vaccine choices and one no vaccine. The vaccines varied by risk of infection, serious illness, protection duration, mild or severe side effects, and population coverage. We used the Qualtrics web-based survey platform. It randomly assigned each respondent one of 6 blocks of choice sets. Respondents saw the same block of questions as initially assigned to them in each survey wave.

The choice sets were generated using a D-efficient design [35] from the NGene software package (ChoiceMetrics) [36]. They were calibrated such that an overall view of each respondent’s vaccine preferences over key vaccine attributes could be established, within credible ranges. Thus, they give us an overall view of COVID-19 vaccine preferences, rather than asking about specific vaccines that were in development. Each of the 4 question blocks is balanced such that a raw count of the number of vaccines chosen out of 6 is roughly comparable between respondents. For more details on the questions, see Hess et al [7].

Discrete, stated choice surveys are widely used in health due to their effectiveness in providing an accurate and fine-scale measure of individuals’ preferences [7,34]. While stated survey measures of sentiment, willingness, or intention to vaccinate are also valid approaches used in the literature (as covered in the introduction), the strengths of a discrete choice experiment mean it is highly suited to understanding vaccine preferences within a specific case study using hypothetical but realistic vaccine options. This is particularly important as the vaccines were still under development and their attributes were not clear. Hence, we could also not measure real vaccine uptake during this period [22].

For each wave, we sum the number of times respondent i selects a vaccine option across the 6 choice scenarios. We then classify individuals into 3 vaccine uptake categories, which we denote as resistant (vaccine chosen 0 times), hesitant (vaccine chosen 1 to 5 times), and provaccine (vaccine chosen 6 of 6 times). As noted in the previous paragraph, there may be some differences between the 4 choice blocks that could drive different counts between individuals, but these should not be significant and are unlikely to affect our classification of respondents into 3 categories (this classification approach reduces the influence of any minor measurement error across the blocks). Respondents also saw the same choice block in each wave, so differences between waves, within individuals, are not due to changes in questions.

In the New Zealand survey of Hess et al [7] in wave 3 only, we asked about the frequency of social media use, by platform, in the last 6 months. This period roughly equates with the time between the first and third survey waves, so that we can measure the association between use and changes in vaccination uptake preferences between survey waves. Platforms included are Facebook, Instagram, Twitter, and TikTok. We use dummy coding, with anyone using the platform at least once a week being coded as a user.

We asked participants about their trust in various sources for information about COVID-19 vaccines. We test their moderating effect on social media and vaccine uptake. They were measured on a 5-point Likert scale, and we coded them as dummy variables of trust (1) versus neutral or distrust (0).

We pool our data across the 3 waves, including social media use by type, a rich set of covariates, and time fixed effects. The original wave 1 sample included was representative by age and gender; however, due to attrition, the wave 3 sample needs some reweighting. We reweight for all analyses on the basis of age, gender, and ethnicity from the 2018 census [37,38]. We drop 110 observations with missing data on the variables we use in the modeling. As we collected social media use in wave 3 only, we are left with a balanced panel across the 3 waves of 257.

### Ethical Considerations

This project was granted ethics approval for research by the Waikato Management School Ethics Committee (application WMS 20/68). The panel was recruited using the Qualtrics survey company, and respondents were provided a small financial incentive by Qualtrics to participate in the sample. Participants provided their informed consent to participate in the study and had the option to withdraw their data at any time. All data were anonymized by Qualtrics before being sent to the research team to protect the privacy and confidentiality of our research participants.

### Empirical Modeling

We are interested in understanding three main points from the data as follows: (1) the association between social media use (by platform) and an individual becoming less (or more) provaccine over time; (2) the overall association of social media use (by platform) with likely vaccine uptake, and (3) the moderating influence of trust in information sources and vaccine uptake.

To understand the first point, we estimate simple Logit models for each transition between waves. This parsimonious modeling approach allows us to estimate the association between social media use and an increase/decrease in likely vaccine uptake over time, at an individual level. Our dependent variable is a binary indicator $L_{ijk}=1$ for when individual i transitioned to a lower vaccine uptake group between waves j and k, and $H_{ijk}$ indicating if individual i transitioned to a higher vaccine uptake. We include the full set of control variables. We model the transitions separately for the different starting groups in wave j because these groups are fundamentally different from each other. For example, we model $L_{ijk}$ for provaccine individuals, for each wave. This allows us to understand whether social media is associated with staying provaccine or becoming less provaccine between waves j and k. If we had not separated these groups out, we would have implicitly treated transitions from being provaccine to hesitant the same as moving from hesitant to resistant.

For the latter 2 points, we estimate a PPO model [33] on the ordinal dependent variable of stated vaccine uptake of resistance through to provaccine. This model can give us an overall estimate of the association between social media platforms and vaccine uptake. However, it will not give us as clear a picture of transition over time as the Logit approach outlined above.

The PPO is a specific case of a generalized ordered logit (gologit). It allows us to take into account the ordinal nature of our outcome variable, but relax the proportional odds assumption of the more commonly used ordered logit, when needed [39,40]. The proportional odds assumption states that if we estimate a series of cumulative logit models by successively collapsing the ordinal variable into a binary variable (with different cutoffs), the odds ratios for each regressor will be equal across all models (within the limits of random error). When this assumption is violated, ordinal logit models may produce misleading and biased results [40]. Brant [41] developed a test to determine whether the proportional odds assumption holds for a set of variables. In our case, we find the proportional odds assumption to be violated for several covariates and the overall model. Hence, the PPO model allows us to relax the proportional odds assumption when needed, but keep it for the covariates where it is not violated.

An alternative model we could have estimated, which also does not require the proportional odds assumption to hold, is the multinomial logit model [39]. This model runs a series of logit regressions on every possible binary combination of the categorical variable (in our case, {1 vs 2, 1 vs 3, 2 vs 3}). However, this approach fails to account for the inherent ordering of $U_{it}$ and computes a number of unnecessary parameters [40].

Using maximum likelihood, our PPO model estimates the probability that individual i in wave t is resistant, hesitant, or pro, represented by $U_{it}\in \left\{1,2,3\right\}$, respectively:


$$P\left(U_{it}>k\right)=\frac{exp\left(\alpha_{k}+X_{it}\beta 1_{k}+Z_{it}\beta 2\right)}{1+\left[exp\left(\alpha_{k}+X_{it}\beta 1_{k}+Z_{it}\beta 2\right)\right]},k=\left(1,2\right).$$

In our case, we can think of the PPO model as essentially 2 sets of logits, modeling the probability of being in categories 2 and 3 over 1 for the first set of coefficients (k=1), and category 3 over 1 and 2 for the second set of coefficients (k=2). Of course, both logit models are estimated simultaneously, and both are used to calculate predicted probabilities of being in any given category, as represented in the above equation. The coefficients ${\beta 1}_{k}$ for covariates $X_{it}$ vary over k. The coefficients β2 for covariates $Z_{it}$ do not vary over k when they do not violate the proportional odds assumption, reducing the number of coefficients to estimate. Covariates are placed into either $X_{it}$ or $Z_{it}$, depending on the results of Brant tests. These include time-invariant variables, such as social media use, and wave time fixed effects.

To investigate our aim (2) (stated above), we estimate a separate PPO for each social media platform, plus the full set of controls. For aim (3), we interact social media use with trust in different sources of information on vaccines (friends or family, the government, and social media).

## Results

### Descriptive Statistics

We start by presenting summary statistics in Table 1 for the variables we use in our modeling (pooled across the 3 waves). In terms of social media use, most of the sample uses at least one social media platform out of the 4 we questioned them on (188/257, 73.2%). A majority of respondents use Facebook (177/257, 68.9%), with use of Instagram, Twitter, and TikTok being significantly lower at 30.4% (n=78), 15.2% (n=39), and 7% (n=18), respectively. Most respondents trust the government (199/257, 77.4%) and a minority trust their friends and family (84/257, 32.7%) as sources of vaccine information. As discussed in the Data section, our New Zealand sample is an informative context to study vaccine preferences, given the relatively high levels of trust in government, as COVID-19 vaccines were rolled out. Moreover, most respondents distrust social media generally (195/257, 75.9%) as a source of vaccine information. Across the 3 waves, we classify 76.1% (587/771) of responses as belonging to the provaccine uptake group, 18.3% (141/771) of responses as belonging to the vaccine-hesitant uptake group, and 5.6% (43/771) of responses as belonging to the resistant uptake group.

**Table 1.** Summary statistics.

Variable	Values		Total	Observations
	N (%)	Mean (SD)		
Resistant	5.6	—^a^	257	771
Hesitant	18.3	—	257	771
Pro	76.1	—	257	771
Social media user	73.2	—	257	257
Facebook user	68.9	—	257	257
Instagram user	30.4	—	257	257
Twitter user^b^	15.2	—	257	257
TikTok user	7.0	—	257	257
Female	42.9	—	257	257
Male	57.1	—	257	257
Maori and Pacific	5.4	—	257	257
Trusts family or friends	32.7	—	257	257
Trusts government	77.4	—	257	257
Distrusts social media	75.9	—	257	257
University-educated	43.2	—	257	257
Income (NZ $, 000s)^c^	—	46.1 (35)	257	771
Age (years)	—	52.0 (15.7)	257	257

a Not applicable.

b This work was undertaken before the change of ownership and approach at Twitter in 2022. Most users of less-common social media platforms in our sample (TikTok, Twitter and Instagram) also used Facebook. Only 6% of social media users did not use Facebook.

c This variable is measured in New Zealand dollars (NZ $). At the modal time this variable was measured (August 25, 2020), the exchanged rate to US dollars (US $) was NZ $1 = US $0.65,

In Figure 1*,* we use a Sankey flow diagram to visually depict transitions between vaccine uptake groups across the 3 waves. Evidently, there are significant movements between vaccine uptake groups over time, particularly for the hesitant. Overall, we see movement away from hesitant by wave 3, towards provaccine and resistant. There is also a considerable proportion of respondents who remain stable in their vaccine uptake group membership across the 3 waves. Multimedia Appendix 1 contains the transition matrices.

**Figure 1.**
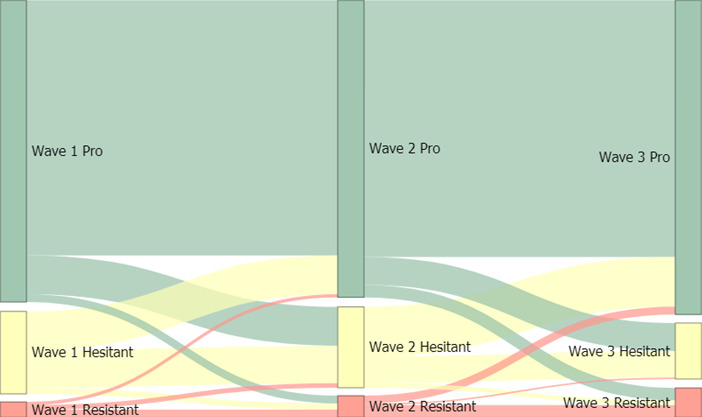
Sankey diagram of individual transitions between vaccine uptake groups across waves.

### Transition Models

In Table 2, we present the marginal effects at the means (MEMs) of social media use on the probability of provaccine individuals moving to a lower vaccine uptake group. These marginal effects show the change in probability for the dependent variable occurring when all other variables are set at their sample means. The first column shows the transition between waves 1 and 2, and the second column represents the transition between waves 2 and 3. Each row represents the MEMs from a model with a full set of controls, and one type of social media platform (or all social media platforms in the case of the first row). The full logit results, including controls, are in Multimedia Appendix 1.

**Table 2.** Marginal effects at the means (MEMs) of social media use on the probability that provaccine individuals decrease uptake between waves. Cluster robust SEs in parentheses. Results have been reweighted on age, gender, and ethnicity.

MEMs for platform use	Dep. variable: decrease in uptake	
	Waves 1 to 2 (n=194), mean (SE)	Waves 2 to 3 (n=191), mean (SE)
Social media use	−0.0187 (0.0678)	−0.109 (0.0879)
Facebook use	0.0161 (0.0583)	−0.156 (0.0829)^a^
Instagram use	−0.0484 (0.0549)	−0.132 (0.0827)
Twitter use	−0.164 (0.0566)^b^	−0.145 (0.0777)^a^
TikTok use	−0.151 (0.0548)^b^	−0.144 (0.0809)^a^

a *P*<.1

b *P*<.01

We can see that Twitter and TikTok are associated with a significantly lower probability of provaccine individuals decreasing uptake between waves 1 and 2 (P<.01). Twitter users are 16.4 percentage points less likely to decrease uptake, and TikTok users are 15.1 percentage points less likely. The MEMs are similar in size for these 2 platforms for the waves 2 to 3 transition (however, the MEMs are only marginally significant). The MEMs are significantly stronger for the waves 2 to 3 transition for general social media, Facebook, and Instagram use. For the waves 2 to 3 transition, provaccine Facebook users are 15.6 percentage points less likely to decrease vaccine uptake (significant at the 10% level). We do not estimate this model for the vaccine-hesitant group moving to resistant, as there are too few such transitions.

We estimate the increase in uptake from hesitant in Table 3. We find statistically significant MEMs of social media use on the probability of positive changes in uptake for the waves 1 to 2 transition. Hesitant social media, Facebook, and Instagram users are 29.6, 23.4, and 45.3 percentage points more likely to increase uptake (respectively) between waves 1 and 2. We find no significant MEMs for the waves 2 to 3 transition. We do not include any other transition models due to sample size and rarity of transitions. For example, only 4 individuals went from hesitant to resistant between waves 1 and 2, making it impossible to model these transitions using our suite of covariates.

**Table 3.** Marginal effects at the means (MEMs) of social media use on the probability that vaccine-hesitant individuals increase uptake between waves. Cluster robust SEs in parentheses. Results have been reweighted on age, gender, and ethnicity.

MEMs for platform use	Dep. variable: increase in uptake	
	Waves 1 to 2 (n=53), mean (SE)	Waves 2 to 3 (n=52), mean (SE)
Social media use	0.296 (0.105)^a^	0.108 (0.157)
Facebook use	0.234 (0.112)^b^	0.0694 (0.151)
Instagram use	0.453 (0.150)^a^	−0.101 (0.266)
Twitter use	0.0264 (0.131)	0.166 (0.179)
TikTok use	0.107 (0.200)	0.0315 (0.265)

a *P*<.01

b *P*<.05

### Partial Proportional Odds Model Results

In Table 4, we present the coefficients for the full PPO results of our base models with pooled data, wave fixed effects, and no interactions. Each pair of columns shows a separate model, varying only by the social media platform dummy included in the model. The first set of columns, (1), is social media use of the specified platform. The left column, labeled 1 versus 2, 3, models being hesitant or provaccine, over being resistant. Hence, a positive coefficient indicates a more provaccine orientation (more likely to be hesitant or provaccine than resistant). The right column, labeled 1, 2 versus 3, models being provaccine, over being hesitant or resistant, with positive coefficients again indicating a more provaccine orientation. Where coefficients are missing, these have been restricted to being the same across both columns, as the proportional odds assumption is not violated. As described in the Methods section, our modeling approach allows us to relax the assumption that covariates have the same impact on preferences across different transitions. Moreover, our results in Table 4 show how social media use and a range of individual characteristics correlate with our novel vaccine preference groupings derived from discrete choice experiment data.

**Table 4.** Full partial proportional odds (PPO) modeling results with time fixed effects, excluding interactions. Each pair of columns represents a separate model, varying only by type of Social media user, in the column heading. Cluster robust SEs in parentheses, clustered at the individual level. Results have been reweighted on age, gender, and ethnicity. 1 versus 2, 3 shows the model predicting the likelihood of being 2 (hesitant) or 3 (pro), over 1 (resistant), where a positive number shows more likely to be in 2 or 3. No coefficient in 1, 2 versus 3 means the model is restricted to assume the same coefficient across both columns, as the proportional odds assumption is not violated for that variable. Wave FE (fixed effect) is a fixed effect, coded as a dummy variable for that wave.

	Dependent variable: vaccine preference, where resistant is 1 and pro is 3									
	(1) Social media, mean (SE)		(2) Facebook, mean (SE)		(3) Instagram, mean (SE)		(4) Twitter, mean (SE)		(5) TikTok, mean (SE)	
	1 versus 2, 3	1, 2 versus 3 (n=257, Observations=771)	1 versus 2, 3	1, 2 versus 3 (n=257, Observations=771)	1 versus 2, 3	1, 2 versus 3 (n=257, Observations=771)	1 versus 2, 3	1, 2 versus 3 (n=257, Observations=771)	1 versus 2, 3	1, 2 versus 3 (n=257, Observations=771)
Social media user	0.265 (0.292)	—^a^	0.249 (0.287)	—	3.760 (0.764)^b^	1.274 (0.410)^b^	14.99 (0.436)^b^	1.024 (0.576)^c^	0.377 (0.587)	—
Income	0.00796 (0.00404)^c^	—	0.00815 (0.00410)^d^	—	0.0544 (0.0194)^b^	0.00855 (0.00438)^c^	0.00820 (0.00392)^d^	—	0.00835 (0.00398)^d^	—
University	−0.352 (0.348)	—	−0.358 (0.349)	—	−0.361 (0.304)	—	−0.250 (0.291)	—	−0.335 (0.358)	—
Age (years)	−0.0115 (0.00984)	—	−0.0118 (0.00975)	—	0.00885 (0.0101)	—	−0.00426 (0.00898)	—	−0.00967 (0.00851)	—
Male	0.0593 (0.309)	—	0.0652 (0.310)	—	0.284 (0.299)	—	−0.0757 (0.297)	—	0.00144 (0.277)	—
Māori and Pacific	−0.935 (0.515)^c^	—	−0.944 (0.515)^c^	—	−0.947 (0.446)^d^	—	−0.847 (0.442)^c^	—	−0.999 (0.512)^c^	—
Trust government	0.841 (0.298)^b^	—	0.853 (0.298)^b^	—	2.465 (0.562)^b^	0.895 (0.292)^b^	0.849 (0.263)^b^	—	0.820 (0.292)^b^	—
Trust family or friends	−0.126 (0.352)	—	−0.136 (0.349)	—	−1.399 (0.552)^d^	−0.110 (0.313)	−0.139 (0.302)	—	−0.178 (0.323)	—
Wave 2 FE	0.146 (0.259)	—	0.146 (0.259)	—	0.183 (0.275)	—	0.169 (0.263)	—	0.153 (0.260)	—
Wave 3 FE	−0.618 (0.360)^c^	0.340 (0.256)	−0.618 (0.360)^c^	0.340 (0.256)	−0.618 (0.406)	0.352 (0.243)	−0.584 (0.390)	0.338 (0.259)	−0.604 (0.363)^c^	0.348 (0.254)
Intercept	3.128 (0.883)^b^	0.726 (0.775)	3.153 (0.882)^b^	0.752 (0.770)	−0.255 (0.846)	−0.644 (0.655)	2.704 (0.665)^b^	0.390 (0.532)	3.229 (0.850)^b^	0.826 (0.670)

a Not applicable.

b *P*<.01.

c *P*<.1

d *P*<.05

Looking across the models, we find that Instagram and Twitter use have a statistically significant positive relationship with vaccine uptake. On the other hand, at least one of the 4 types of social media, Facebook and TikTok use, did not have significant effects. We present the MEMs for social media of these models in Table 5. Here, we see highly statistically significant associations for Instagram and Twitter. Specifically, Instagram is associated with an 11.8 percentage point decrease in the likelihood of being resistant and a 20.3 percentage point increase in the likelihood of being provaccine. The direction is the same for Twitter, albeit with lower marginal effects. It is worth noting again that Twitter and TikTok use is relatively uncommon in our sample (Table 1).

In terms of demographic controls in Table 5, we see that income has a positive and significant association with vaccine uptake. University education, age, and gender do not have a statistically significant association with uptake level. Māori or Pacific ethnicity has a negative association with vaccine uptake, at the 5% or 10% level. Towards the bottom of Table 4, we see that the survey wave fixed effects have little predictive power on vaccine uptake. Wave 3 is associated with a lower chance of being vaccine resistant, with significance at the 10% level for 3 of the 5 models.

**Table 5.** Average marginal effects (marginal effects at the mean) of social media use on being in different vaccine uptake categories (from the models in Table 5). Cluster robust standard errors in parentheses, calculated using the delta-method. Results have been reweighted on age, gender, and ethnicity.

Platform	Resistant, mean (SE)	Hesitant, mean (SE)	Pro, mean (SE)
Social media	−0.0120 (0.0140)	−0.0362 (0.0408)	0.0481 (0.0545)
Facebook	−0.0112 (0.0134)	−0.0338 (0.0396)	0.0450 (0.0527)
Instagram	−0.118 (0.0205)^a^	−0.0858 (0.0563)	0.203 (0.0591)^a^
Twitter	−0.0583 (0.0144)^a^	−0.0967 (0.0702)	0.155 (0.0699)^b^
TikTok	−0.0145 (0.0203)	−0.0485 (0.0704)	0.0630 (0.0904)

a *P*<.01.

b *P*<.05

### Trust Interactions in PPO Models

Next, we look at the trust variables. For the base models in Table 5, we see that the coefficient on trust in government for information about vaccines is positive and strongly statistically significant, as expected. Trust in family or friends for vaccine information has a negative but statistically insignificant relationship with uptake, except for the Instagram model, where it is negative and significant for the 1 versus 2, 3 component of the model. We exclude distrust in social media from the model as it leads to within-sample predictions of negative probabilities for a handful of observations (a drawback of PPO models [40]).

Next, we interact with trust in 2 key information sources (government, and friends and family) on vaccines, with social media type in our base PPO models. This analysis is to test whether trust in information sources has a mediating role in how social media use is associated with vaccine uptake.

We might expect that social media use will be associated with a higher likelihood of vaccine uptake for those who trust the government for vaccine information (compared with not trusting), given the use of social media by the government to inform the public about COVID restrictions. Finally, we expect social media users will be less likely to take the vaccine if they trust their friends or family for vaccine information (compared with not trusting), given that friends or family may share vaccine information on social media and are a less reliable source of information. To test these hypotheses, we follow the same approach as before and re-estimate the models from Table 5, with just the interaction of interest. We present the MEMs for these interactions in Table 6.

Let us first consider general social media use and trust in government, in the top left of the table. We see that social media use for those who trust the government is associated with a 4.7% lower probability of being resistant, which is statistically significant at the 5% level. However, none of the other marginal effects is significant, nor is the difference between the marginal effects for those who trust the government for vaccine information and those who do not. There is a similar result for Facebook users.

**Table 6.** Marginal effects at the mean (MEMs) for the interactions between social media use and trust in sources of information regarding vaccines. Cluster robust SEs in parentheses, calculated using the delta method. Results have been reweighted on age, gender, and ethnicity.

MEMs	Trust in government			Trust in friends and family		
	Resistant	Hesitant	Pro	Resistant	Hesitant	Pro
Social media, mean (SE)						
Trust=0	−0.00527 (0.0420)	−0.00823 (0.0651)	0.0135 (0.107)	−0.0222 (0.0167)	−0.0668 (0.0481)	0.0891 (0.0638)
Trust=1	−0.0470 (0.0184)^c^	−0.00534 (0.0523)	0.0524 (0.0633)	0.0227 (0.0189)	0.0768 (0.0660)	−0.0995 (0.0840)
Difference significance	—^a^	—	—	Yes^b^	Yes^b^	Yes^b^
Facebook, mean (SE)						
Trust=0	0.0445 (0.0462)	−0.0824 (0.0796)	0.0379 (0.111)	−0.0209 (0.0157)	−0.0635 (0.0459)	0.0844 (0.0606)
Trust=1	−0.0255 (0.0150)^b^	−0.0274 (0.0560)	0.0530 (0.0617)	0.0229 (0.0191)	0.0757 (0.0647)	−0.0986 (0.0829)
Difference significance	—	—	—	Yes^b^	Yes^b^	Yes^b^
Instagram, mean (SE)						
Trust=0	−0.260 (0.0413)^d^	−0.00929 (0.0983)	0.269 (0.104)^c^	−0.0905 (0.0203)^d^	−0.107 (0.0632)^b^	0.197 (0.0696)^d^
Trust=1	−0.0455 (0.0189)^c^	−0.133 (0.0599)^c^	0.179 (0.0625)^d^	−0.0653 (0.0325)^c^	−0.175 (0.0708)^c^	0.240 (0.0875)^d^
Difference significance	Yes^d^	Yes^b^	—	—	—	—
Twitter, mean (SE)						
Trust=0	−0.117 (0.0315)^d^	−0.306 (0.0849)^d^	0.424 (0.0887)^d^	−0.0525 (0.0148)^d^	−0.00257 (0.0994)	0.0551 (0.0990)
Trust=1	−0.0393 (0.0104)^d^	−0.0104 (0.0751)	0.0496 (0.0763)	−0.0746 (0.0189)^d^	−0.196 (0.0770)^c^	0.270 (0.0815)^d^
Difference significance	Yes^d^	Yes^d^	Yes^d^	—	Yes^b^	Yes^b^
TikTok, mean (SE)						
Trust=0	−0.106 (0.0280)^d^	−0.302 (0.0588)^d^	0.408 (0.0693)^d^	−0.0522 (0.0138)^d^	−0.0571 (0.106)	0.109 (0.107)
Trust=1	−0.0364 (0.00972)^d^	0.127 (0.116)	−0.0906 (0.116)	−0.0384 (0.0267)	0.0245 (0.157)	0.0139 (0.156)
Difference significance	Yes^d^	Yes^d^	Yes^d^	—	—	—

a Not applicable.

b *P*<.1

c *P*<.05

d *P*<.01

Instagram, Twitter, and TikTok users have higher probabilities of being pro and lower probabilities of being hesitant or resistant, for both individuals who trust the government and those who do not. The MEMs of social media use on those who trust the government and those who do not are counterintuitive. For example, Twitter use is associated with a 11.7 percentage point decrease in the probability of being resistant, for those who do not trust the government. On the other hand, Twitter use is associated with only a 3.9 percentage point decrease in being resistant for those who do trust the government. This is a statistically significant difference. Instagram and TikTok use shows largely similar patterns to the Twitter example mentioned here. Thus, Twitter, Instagram, and TikTok use is associated more positively with vaccine uptake for those who do not trust the government, compared with those who do trust the government.

The right side of Table 6 shows the MEMs for interactions between social media use and trust in friends and family. We might expect those who trust friends and family for vaccine information to be more susceptible to misinformation on social media. We see this for social media users in general in the top rows; social media users who trust friends and family are more likely to be resistant and hesitant, and less likely to be pro, at the 10% level. This finding is true for Facebook users as well. There are no significant differences for Instagram or TikTok users. However, we see the opposite for Twitter users. Twitter users who trust their friends and family are more likely to be pro and less likely to be hesitant compared with those who do not trust their friends and family, again at the 10% level of significance.

## Discussion

In this paper, we have demonstrated a novel approach to analyzing stated preference data. Specifically, we categorize individuals as resistant, hesitant, or pro, based on the number of times they chose a vaccine option in a set of 6 stated choices. Our stated choice method (discrete choice experiment) is a well-established means of understanding individual vaccine preferences; our means of analyzing such data adds another method to understanding vaccine orientation. We investigate the association between individual choices and social media use over time using both a Logit and PPO model, and we use the PPO model to understand the moderating role of trust in sources of information. Our data were collected during vaccine development and initial rollout, between August 2020 and April 2021. Our context in New Zealand had a high trust in the government at the time.

We find a positive or neutral association between social media use and vaccine uptake. In our transition modeling, we see some evidence that Facebook, Twitter, and TikTok users who are pro are less likely to become hesitant or resistant. Facebook and Instagram users who are hesitant are more likely to become pro. Over the 3 survey waves, we find a positive association between Instagram and Twitter and being more likely to uptake the vaccine.

Our results on trust in information sources provide some more interesting details. As expected, we find a strong positive association between trust in government for vaccine information and being more provaccination, shown in our main PPO results in Table 4. However, we provide some additional evidence that some social media platforms may have a more positive effect on vaccine uptake preferences for those who do not trust the government. Our Table 5 results show Instagram, Twitter, and TikTok use is more positively associated with being more provaccination for those who do not trust the government, compared with those who trust the government. This finding points to a potentially positive role for some social media platforms. Potentially good information from trusted, non-government sources could reach those who lack trust in the government but are still open to being convinced on the merits of vaccination.

These findings provide a marginally positive picture for social media and vaccine uptake. However, given the concerns from the wider literature outlined in our introduction, we urge caution in how our results are interpreted. While social media could help increase vaccine uptake, our data do not strongly refute the potential for social media to also decrease vaccine uptake. Social media still has just as significant potential to spread bad information about vaccination to susceptible individuals. Indeed, the spread of misinformation via social media and its impact on vaccine hesitancy has been a key concern in the literature [22].

There are several reasons why we may not have found a negative association between social media use and vaccine preferences. First, a limitation of our study is the dataset itself. The social media question is only in the third survey wave, and asks about social media use over the previous 6 months. It would have been preferable to include social media use in each survey wave and ask about social media use over a shorter timeframe. Future research would also ideally cover other social media platforms that may spread misinformation, such as Telegram and YouTube. However, our mixed results by platform align with previous work that shows different social media platforms have varying effects on the spread of vaccine misinformation [29]. Despite these limitations, we argue that our novel analysis of a stated choice survey and the panel nature of the dataset still demonstrate a useful contribution.

Second, we undertake a web-based survey, with potential for sample selection bias. It seems likely that individuals with conspiracy beliefs will be less likely to undertake a survey from University researchers. Hence, we may have missed those at the most extreme end of resistance. As such, a survey may not be the best approach to understand the extremely resistant. Data pulled directly from the use of social media platforms may help in this regard [19]. Of course, analyzing such data can give a better idea of social media users, but it does not include a control of non-social media users, and may not be as clear in providing information about individual vaccine preferences.

Third, in the context of high government trust at the time in New Zealand, we find low levels of vaccine resistance of 4 to 7 percent, which is relatively low by international comparison [7]. This low proportion makes it impossible to identify the reasons behind the increasing levels of resistance we observed over time, without a much larger sample size. Thus, we acknowledge that other contexts are likely to have much higher potential for misinformation on social media, having a greater impact on levels of hesitancy or resistance. For instance, previous work in the United States using panel data found that social media was associated with higher levels of vaccine hesitancy during the COVID-19 pandemic [28]. Furthermore, we do observe polarization, with increasing levels of individuals in both the resistant and pro categories over time.

We note that a limitation of our data is that it cannot be interpreted as causal, hence, we frame our findings as associations between social media use and vaccine preferences. We collected social media use for individuals in survey wave 3, over the last 6 months. This period coincides approximately with the length of time between the first and third survey waves. However, this is second-best panel data; first-best would include recent social media use in each wave, meaning we could track longitudinally how changes in social media use change vaccine preferences. This would allow us to be closer to finding causal relationships, though we are not sure how much social media use changes over time, and therefore, if such an approach would allow us enough variation. There may still be potential endogeneity issues with such an approach. We also point out that the transition modelling gets us closer to a causal interpretation, as opposed to cross-sectional associations, as we see whether changes in vaccine preferences are associated with social media use between observations.

Other future research could use a similar stated preference panel data method to track the evolution of vaccine preferences. However, it would be helpful if it included more detailed questions on social media use and other sources of vaccine information. Our study highlights the need to collect such vaccine preference data at the crucial point where new vaccines are developed and rolled out, so that we can study how preferences form. Thus, opportunities should be explored to collect such data on new COVID vaccines and other new vaccines in development.

While our study was overall positive in terms of social media use and vaccine uptake, we contribute to a literature with a mixed picture, as outlined in the introduction. The concerning potential for polarization to continue to increase is still ever present. The downsides are large, including both worsening public health as well as increasingly extreme political conflict based on disagreement over basic facts. Whether naive or bad actors are spreading misinformation, it is a pressing issue to both better understand the problem and potential solutions.

## Supplementary material

10.2196/66081 Multimedia Appendix 1 Additional modeling results.

## Abbreviations

MEM: marginal effects at the mean
PPO: partial proportional odds
