# Supplementary material for: Social Media and the Evolution of Vaccine Preferences During the COVID-19 Pandemic: Discrete Choice Experiment
Source: JMIR Infodemiology. 2025 May 28;5:e66081. doi: 10.2196/66081 (PMC12165271; doi:10.2196/66081)
Supplement: Multimedia Appendix 1 [file infodemiology-v5-e66081-s001.docx]

**Appendix**

**Transition matrices for vaccine uptake across the three waves.**

Please note that the percentages reflect the proportion of respondents who were in the row uptake category in wave 1 (or wave 2) and are now in the column uptake category in wave 2 (or wave 3).

Table S1. Transition matrices of vaccine uptake across the waves.

|  | **Wave 2 Vaccine Uptake** | | |  |
| --- | --- | --- | --- | --- |
| **Wave 1 Uptake** | Pro | Hesitant | Resistant | Total (wave 1) |
| Pro | 84.5% (164) | 12.9% (25) | 2.6% (5) | 75.5% (194) |
| Hesitant | 47.2% (25) | 45.3% (24) | 7.5% (4) | 20.6% (53) |
| Resistant | 20.0% (2) | 30.0% (3) | 50.0% (5) | 3.9% (10) |
| Total (wave 2) | 74.3% (191) | 20.2% (52) | 5.4% (14) | 100.0% (257) |
|  | **Wave 3 Vaccine Uptake** | | |  |
| **Wave 2 Uptake** | Pro | Hesitant | Resistant | Total (wave 2) |
| Pro | 86.4% (165) | 9.4% (18) | 4.2% (8) | 74.3% (191) |
| Hesitant | 61.5% (32) | 32.7% (17) | 5.8% (3) | 20.2% (52) |
| Resistant | 35.7% (5) | 7.1% (1) | 57.1% (8) | 5.4% (14) |
| Total (wave 3) | 78.6% (202) | 14.0% (36) | 7.4% (19) | 100.0% (257) |

*Note: Number of respondents (N) are in parentheses.*

**Full logit results for pro-vaccine decrease in uptake transition models.**

|  | *Dependent variable:* A downward shift in vaccine uptake group between waves (0,1) | | | | | | | | | |
| --- | --- | --- | --- | --- | --- | --- | --- | --- | --- | --- |
|  | (1) | | (2) | | (3) | | (4) | | (5) | |
|  | Social Media | | Facebook | | Instagram | | Twitter | | Tik Tok | |
|  | Wave transition | | | | | | | | | |
|  | 1 to 2 | 2 to 3 | 1 to 2 | 2 to 3 | 1 to 2 | 2 to 3 | 1 to 2 | 2 to 3 | 1 to 2 | 2 to 3 |
| Social Media User | -0.145 | -0.917 | 0.131 | -1.306^**^ | -0.392 | -1.280 | -1.983^*^ | -1.620 | -1.978^*^ | -1.684 |
|  | (0.508) | (0.664) | (0.488) | (0.637) | (0.461) | (0.833) | (1.025) | (1.105) | (1.183) | (1.272) |
| Income | -0.00172 | -0.00914 | -0.00173 | -0.0114 | -0.00185 | -0.0127 | -0.00361 | -0.00831 | -0.00416 | -0.0101 |
|  | (0.00682) | (0.0118) | (0.00682) | (0.0127) | (0.00693) | (0.0119) | (0.00654) | (0.00978) | (0.00688) | (0.00998) |
| University | 0.000077 | 0.554 | 0.00681 | 0.619 | 0.0160 | 0.785 | -0.0523 | 0.402 | -0.110 | 0.277 |
|  | (0.529) | (0.695) | (0.522) | (0.723) | (0.527) | (0.700) | (0.479) | (0.699) | (0.484) | (0.750) |
| Age | 0.0117 | 0.00471 | 0.0131 | 0.00164 | 0.00637 | -0.0115 | -0.00123 | -0.00720 | -0.00116 | -0.00723 |
|  | (0.0142) | (0.0153) | (0.0140) | (0.0154) | (0.0137) | (0.0177) | (0.0144) | (0.0151) | (0.0140) | (0.0158) |
| Male | -0.126 | -0.627 | -0.0921 | -0.675 | -0.140 | -0.666 | 0.220 | -0.210 | 0.00651 | -0.378 |
|  | (0.508) | (0.581) | (0.513) | (0.579) | (0.523) | (0.583) | (0.508) | (0.600) | (0.495) | (0.550) |
| Māori and Pacific | -0.162 | 1.868^**^ | -0.109 | 1.978^***^ | -0.190 | 1.984^**^ | -0.172 | 1.722^**^ | -0.110 | 1.763^**^ |
|  | (1.003) | (0.759) | (1.029) | (0.751) | (1.060) | (0.809) | (1.056) | (0.694) | (1.061) | (0.689) |
| Trust Government | -0.279 | -1.885^***^ | -0.285 | -1.944^***^ | -0.281 | -2.075^***^ | -0.609 | -1.804^***^ | -0.574 | -1.772^***^ |
|  | (0.588) | (0.630) | (0.585) | (0.630) | (0.579) | (0.641) | (0.543) | (0.533) | (0.514) | (0.518) |
| Trust Family/Friends | 0.262 | -0.149 | 0.248 | -0.111 | 0.319 | -0.0454 | 0.437 | -0.0840 | 0.385 | -0.00265 |
|  | (0.495) | (0.706) | (0.496) | (0.675) | (0.494) | (0.719) | (0.469) | (0.686) | (0.467) | (0.653) |
| Intercept | -1.919^*^ | -0.0824 | -2.215^**^ | 0.362 | -1.630 | 0.637 | -0.999 | -0.0740 | -0.959 | 0.00878 |
|  | (1.015) | (1.234) | (1.011) | (1.189) | (0.998) | (1.337) | (0.983) | (1.105) | (0.953) | (1.160) |
| *N* | 194 | 191 | 194 | 191 | 194 | 191 | 194 | 191 | 194 | 191 |

Cluster robust standard errors in parentheses; ^*^ *p* < 0.10, ^**^ *p* < 0.05, ^***^ *p* < 0.01

**Full logit results for vaccine-hesitant increase in uptake transition models.**

|  | *Dependent variable:* An upward shift in vaccine uptake group between waves (0,1) | | | | | | | | | |
| --- | --- | --- | --- | --- | --- | --- | --- | --- | --- | --- |
|  | (1) | | (2) | | (3) | | (4) | | (5) | |
|  | Social Media | | Facebook | | Instagram | | Twitter | | Tik Tok | |
|  | Wave transition | | | | | | | | | |
|  | 1 to 2 | 2 to 3 | 1 to 2 | 2 to 3 | 1 to 2 | 2 to 3 | 1 to 2 | 2 to 3 | 1 to 2 | 2 to 3 |
| Social Media User | 1.954^**^ | 0.595 | 1.519^*^ | 0.381 | 3.077^**^ | -0.550 | 0.167 | 0.996 | 0.721 | 0.175 |
|  | (0.793) | (0.867) | (0.808) | (0.828) | (1.152) | (1.451) | (0.827) | (1.188) | (1.476) | (1.476) |
| Income | 0.0195 | 0.00942 | 0.0193 | 0.00896 | 0.0196 | 0.00956 | 0.0160 | 0.00640 | 0.0175 | 0.00896 |
|  | (0.0138) | (0.0173) | (0.0141) | (0.0172) | (0.0153) | (0.0169) | (0.0137) | (0.0170) | (0.0152) | (0.0184) |
| University | -1.106 | 0.105 | -1.000 | 0.0902 | -0.821 | 0.105 | -1.006 | 0.0675 | -0.967 | 0.0831 |
|  | (0.862) | (0.766) | (0.853) | (0.768) | (0.855) | (0.776) | (0.796) | (0.808) | (0.844) | (0.776) |
| Age | -0.0165 | 0.0338 | -0.0292 | 0.0306 | -0.00522 | 0.0154 | -0.0302 | 0.0291 | -0.0261 | 0.0269 |
|  | (0.0263) | (0.0285) | (0.0299) | (0.0277) | (0.0261) | (0.0387) | (0.0295) | (0.0280) | (0.0292) | (0.0278) |
| Male | -1.018 | 1.694^*^ | -0.786 | 1.717^**^ | 0.175 | 1.681^*^ | -0.686 | 1.844^**^ | -0.761 | 1.710^**^ |
|  | (0.705) | (0.858) | (0.705) | (0.855) | (0.767) | (0.851) | (0.706) | (0.886) | (0.708) | (0.848) |
| Māori and Pacific | -0.0582 | 0.450 | -0.298 | 0.309 | -0.154 | -0.00832 | -0.119 | 0.162 | -0.333 | 0.0915 |
|  | (0.788) | (1.088) | (0.865) | (1.047) | (0.898) | (1.147) | (0.942) | (1.002) | (0.976) | (1.103) |
| Trust Government | -1.385^*^ | 0.829 | -1.285^*^ | 0.866 | -0.792 | 0.966 | -1.414^*^ | 0.998 | -1.621 | 0.909 |
|  | (0.753) | (0.863) | (0.749) | (0.864) | (0.866) | (0.917) | (0.811) | (0.832) | (1.046) | (0.880) |
| Trust Family/Friends | -2.665^**^ | 1.331 | -2.864^**^ | 1.370 | -2.773^**^ | 1.549 | -2.402^*^ | 1.529 | -2.673 | 1.454 |
|  | (1.314) | (0.957) | (1.381) | (0.961) | (1.184) | (1.039) | (1.253) | (0.994) | (1.611) | (0.950) |
| Intercept | 1.150 | -3.942^*^ | 2.090 | -3.648^*^ | 0.410 | -2.608 | 3.165 | -3.516^*^ | 3.112 | -3.255^*^ |
|  | (1.559) | (2.102) | (1.739) | (2.003) | (1.693) | (2.476) | (1.947) | (1.918) | (1.903) | (1.878) |
| *N* | 53 | 52 | 53 | 52 | 53 | 52 | 53 | 52 | 53 | 52 |

Cluster robust standard errors in parentheses; ^*^ *p* < 0.10, ^**^ *p* < 0.05, ^***^ *p* < 0.01
